# Improving the Cycling Stability of Next-Generation Si Anode Batteries Using Polymer Coatings

**DOI:** 10.3390/ma18245630

**Published:** 2025-12-15

**Authors:** Ki Yun Kim, Seong Soo Kang, Young-Pyo Jeon, Jin-Yong Hong, Jea Uk Lee

**Affiliations:** 1 Department of Materials Science Engineering, Kyung Hee University, 1732 Deogyeong-daero, Giheung-gu, Yongin-si 17104, Republic of Korea; 12rldbs@khu.ac.kr (K.Y.K.); gksdud09@khu.ac.kr (S.S.K.); 2 Hydrogen & C1 Gas Research Center, Korea Research Institute of Chemical Technology (KRICT), 141 Gajeong-ro, Yuseong-gu, Daejeon-si 34114, Republic of Korea; ypjeon@krict.re.kr; 3 Advanced Materials and Chemical Engineering, University of Science and Technology (UST), 217 Gajeong-ro, Yuseong-gu, Daejeon-si 34113, Republic of Korea

**Keywords:** lithium-ion batteries, silicon, dopamine, conductive, polymer

## Abstract

Silicon is widely recognized as a next-generation anode owing to its exceptional theoretical capacity, yet its practical deployment in lithium-ion batteries is constrained by severe volume expansion, particle fracture, loss of electrical percolation, and solid electrolyte interphase layer instability. Polymer-based strategies have emerged as accessible solutions to engineer extensive volume changes and interfacial compatibility, while preserving pathways for charge transport. Viscoelastic polymer binders dissipate stress, catechol-inspired chemistries strengthen adhesion and tailor interphases, and conductive polymers can function simultaneously as binder, electronic additive, and artificial SEI. This review describes these approaches through a structure–process–performance perspective, emphasizing practically relevant metrics, such as initial capacity, initial Coulombic efficiency, and long-term cycling stability. We organize the main section into (i) dopamine-derived interfacial engineering, (ii) self-healing three-dimensional network binders, and (iii) conductive-polymer-based designs. In the last section, we articulate the functional requirements of polymers in silicon anodes, outline the ideal structural designs, and provide forward-looking avenues for future lithium-ion battery anode research.

## 1. Introduction

The rise of electric vehicles and fast-charging electronic devices is pushing lithium-ion batteries beyond the performance limits achievable with today’s electrode chemistries [1,2]. Graphite-based anodes, while inexpensive and stable, are fundamentally limited by a theoretical capacity of 372 mAh g^−1^ and exhibit rate/temperature sensitivities that constrain fast-charge protocols [3,4,5]. Silicon (Si), by contrast, provides an order-of-magnitude higher theoretical capacity (up to 4200 mAh g^−1^) and a low lithiation potential (~0.2 V vs. Li/Li^+^), promising substantial gains in gravimetric/volumetric energy density and cost per kWh when blended or substituted into graphite frameworks [6,7]. This opportunity is counterbalanced by well-known challenges, ~300% volume expansion upon lithiation, loss of percolated electron pathways, and unstable solid–electrolyte interphase (SEI) growth, that elevate impedance and consume cyclable Li, especially in thick, industrial electrodes [8,9]. Unlike graphite, which forms a stable passivating layer, the SEI on silicon is mechanically unstable due to the large volume fluctuations during cycling. The repetitive expansion and contraction cause the rigid SEI layer to fracture, exposing fresh silicon surfaces to the electrolyte. This leads to continuous electrolyte decomposition and the reformation of a thick, highly resistive SEI layer, which hinders lithium-ion transport and accelerates capacity fading. These realities motivate materials and process innovations that simultaneously manage expansion, stabilize interfaces, and preserve ion/electron transport. The performance of Si anodes is critically evaluated by key metrics such as initial Coulombic Efficiency (ICE, reflecting irreversible Li consumption), rate capability (governing fast-charging kinetics), long-term cycle stability (indicating mechanical integrity and SEI endurance), and energy density (gravimetric capacity). Moreover, these innovations should satisfy under realistic constraints (e.g., ≥3 mAh cm^−2^ areal capacity, controlled swelling, and limited resistance growth).

To address these limitations, two complementary lines of development have emerged. First, nanostructuring Si itself, into nanoparticles, nanowires, porous or hollow/yolk–shell motifs, and engineered secondary microspheres, reduces local stress and shortens diffusion paths, thereby delaying fracture and preserving percolation [10,11,12]. However, the accompanying rise in specific surface area often penalizes initial Coulombic efficiency (ICE), accelerates SEI growth, and lowers tap density, which can erode volumetric energy at practical areal loadings [13]. Second, surface engineering and compositing strategies apply conformal coatings or frameworks around Si using carbonaceous layers (pitch-derived carbon, graphene, carbon nanotubes), conductive polymers, and ultrathin inorganics (e.g., ALD oxides, LiF-forming skins), as well as hybrid interphases that couple these chemistries [14,15,16]. These layers act synergistically as mechanical buffers, electron/ion highways, and SEI directors, stabilizing interfaces while enabling calendering to industrial densities. Among above approaches, construction of a uniform polymer coating on the Si surface is a smart option because it possess high structural flexibility and tunable viscoelasticity, which can tolerate the volume expansion of Si by acting as a soft medium.

Polymers offer three synergistic functions. First, viscoelastic binders dissipate mechanical stress and maintain particle–binder–current collector cohesion during repeated volume change [17]. Second, interfacial chemistries, especially catechol/amine-rich coatings inspired by polydopamine (PDA), improve adhesion to native silanol groups and can template SEI formation toward more inorganic, mechanically robust compositions [18]. Third, conductive polymers (e.g., PEDOT:PSS, polyaniline, polypyrrole, polythiophene and their derivatives) combine electronic transport with compliance, enabling single-component roles as binder, conductive additive, and artificial SEI when properly doped, copolymerized, or functionalized for Si affinity [19,20,21]. While each function of polymer can operate individually, combining them via the diverse functional groups available in polymer systems enables cooperative behavior that accelerates electron and Li^+^ transport, decreases interfacial and bulk resistance, and, in turn, boosts both rate performance and long-term cycling stability of lithium-ion batteries (LIB).

**Figure 1:**
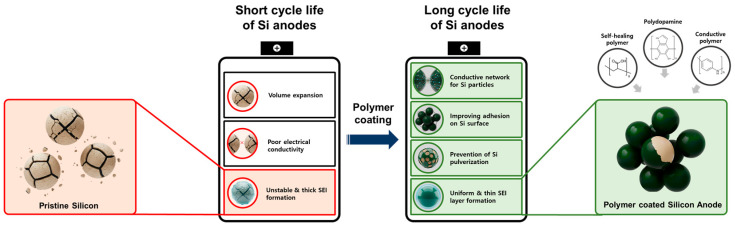
Challenges and strategies for structurally stable Si anodes by employing polymer coatings.

This review paper adopts a structure–process–performance perspective to evaluate polymer strategies across three complementary classes: (i) dopamine-derived interfacial engineering (PDA coatings, carbonized PDA shells, catechol-functional binders, and hybrids with graphene/carbon nanotubes); (ii) self-healing network binders that employ reversible chemistries (dynamic covalent bonds, supramolecular interactions) to recover cohesion and suppress crack propagation of Si anodes; and (iii) conductive polymer-based designs that integrate passivation and transport, including ultrathin artificial-SEI layers grown by in situ electro-polymerization and soft/hard dual-layers that tune Li^+^ flux while buffering stress (Figure 1).

In this review, we present state-of-the-art research on polymer-coated Si anodes, highlighting studies of academic and industrial significance from both nanomaterials-science and battery device-engineering perspectives. Accordingly, we first focus the molecular structure design and material synthesis routes emphasized in each study. It is followed by the numerical comparison of the battery performance (initial capacity, initial Coulombic efficiency (ICE), and long-term cycle stability) achieved by the material combinations. We then discussed the academic novelties of the selected works, their methodological and practical limitations, and potential improvement and translation to realistic operating regimes.

## 2. Bio-Inspired Dopamine Chemistry in Multifunctional Silicon Anode Systems

Dopamine and its polymerized form, PDA, have emerged as versatile materials for interfacial engineering of silicon anodes due to their strong adhesion, abundant functional groups, and bio-inspired chemistry [22,23]. Dopamine replicates the repeating catechol-amine structure found in 3,4-dihydroxy-L-phenylalanine, a component of the mussel foot protein, known for its exceptional adhesion to nearly all organic and inorganic surfaces [18]. Early studies primarily focused on PDA coatings and carbonization to enhance electrical conductivity and mechanical robustness, while recent advances have extended its role into multifunctional binder systems and hybrid nanostructures. These dopamine-based strategies not only buffer the severe volume expansion of Si during lithiation but also improve interfacial stability, ionic transport, and electrode cohesion. This is primarily achieved because the catechol/amine groups provide strong adhesion to Si via hydrogen and coordination bonds, which is critical for electrode cohesion and stability. Furthermore, certain polar functional groups in the PDA derivatives can facilitate ionic transport by offering Li^+^ coordination sites [24]. This section reviews the progressive evolution of PDA-derived coating and binder designs, highlighting their structural concepts, synthesis routes, and electrochemical impacts on silicon-based anodes.

Early in this field, Shi et al. developed a scalable method to synthesize core–shell structured silicon oxide (SiO_x_)/nitrogen-doped carbon composites via PDA coating and carbonization, achieving significant enhancement in electrochemical stability for Li-ion batteries [25]. The composite consists of micron-sized SiOx particles uniformly encapsulated by a ~10–13 nm nitrogen-doped carbon shell derived from the pyrolysis of PDA, which provides both electrical conductivity and mechanical buffering against SiOx volume variation. The synthesis involved thermal evaporation of Si and SiO_2_ (1:1 molar) to form SiOx powder, self-polymerization of dopamine in Tris buffer (pH 8.5) for 24 h, followed by carbonization at 800 °C under N_2_, resulting in uniform, conformal coatings with pyridinic, pyrrolic, and graphitic nitrogen configurations. Electrochemically, the SiOx/C composite showed an initial specific discharge capacity of 2472 mA h g^−1^ and a specific charge capacity of 1610 mA h g^−1^, achieving an initial Coulombic efficiency of 65.1%. In addition, the SiOx/C electrode delivered a reversible capacity of 1514 mAh g^−1^ after 100 cycles at 100 mA g^−1^ and maintained 933 mAh g^−1^ at 2 A g^−1^, outperforming bare SiOx (711 mAh g^−1^) with 85% retention and a near-100% Coulombic efficiency after activation. This study clearly demonstrates that the N-doped carbon shell derived from PDA improved conductivity, buffered structural stress, and facilitated the formation of a stable SEI layer, ensuring high reversibility and long cycle life. Nevertheless, being an early study on PDA-coated silicon anodes, the work reported a relatively low ICE value, which could impede near-term industrial translation.

**Figure 2:**
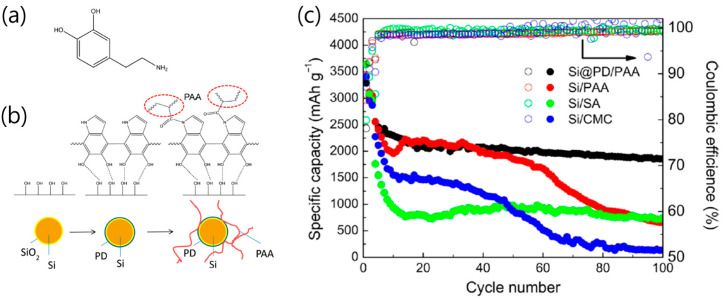
(**a**) Chemical structure of dopamine. (**b**) Graphical instruction of the interaction between different components. (**c**) Cycling performance of Si@PDA/PAA, Si/PAA, Si/sodium alginate (SA) and Si/CMC electrodes (reproduced with permission from [26], copyright 2016, ACS).

Utilizing dopamine’s rich functional diversity, recent strategies extend beyond simple coating and carbonization to (i) construct three-dimensional (3D) network structures via cross-linking and (ii) strengthen interfacial coupling with Si through chemical post-treatments. The first approach is the development of a cross-linked Si anode architecture in which PDA-wrapped Si nanoparticles are chemically bonded with poly(acrylic acid) (PAA) to enhance mechanical integrity and cycle life in LIB (Figure 2a) [26]. The anode material comprised Si nanoparticles coated with a thin (1–2 nm) PDA layer that provided strong hydrogen bonding and reactive amine groups, which further cross-linked with carboxyl groups in PAA to form a robust 3D polymer network (Figure 2b). This structure effectively mitigated Si pulverization and delamination, maintaining electrode cohesion under large volume expansion. The fabrication involved dopamine self-polymerization on Si in alkaline buffer, followed by in situ condensation between PDA and PAA during electrode formation, yielding a mechanically reinforced, conductive composite binder system. Electrochemically, Si@PDA/PAA electrodes achieved a specific capacity of ~1800 mAh g^−1^ after 100 cycles at 1.5 A g^−1^, maintaining electrode integrity even at a high areal loading of 2.0 mg cm^−2^ with 3.69 mAh cm^−2^ and 77% retention after 40 cycles, outperforming Si/PAA and Si/carboxymethyl cellulose (CMC) electrodes (<800 mAh g^−1^) (Figure 2c). Furthermore, the system exhibited an initial Coulombic efficiency of 68.9%, stabilizing above 99% after activation. The academic originality of this work lies in demonstrating that, without high temperature (800–1200 °C) carbonization, a simple in-process wet route, dopamine coating and PAA blending, reinforced adhesion, suppressed cracking, and formed a flexible network that balanced conductivity and elasticity.

The second approach is the post oxidation of a PDA-derived carbon shell on Si nanoparticles to generate lithophilic –OH/–COOH sites markedly improves Si-anode capacity retention and cyclability in LIBs [27]. The material comprised polydisperse Si nanoparticles (approx. 50–100 nm) encapsulated by a turbostratic, conductive carbon shell whose oxygenated functional groups attracted Li^+^ and aided transport to the Si core while buffering volume change. The coating route used dopamine polymerization in Tris buffer, carbonization at 1000 °C to form Si–C, followed by KMnO_4_/H_2_SO_4_ oxidation to install –OH/–COOH groups on the carbon surface (Si-C-AT). Electrochemically, the ICE of bare Si (82.2%), Si-C (83.4%), and Si-C-AT (85.7%) were recorded. The Si-C-AT delivered 1575 mAh g^−1^ after 200 cycles (0.5 C), outperforming Si-C (1261 mAh g^−1^) and bare Si (961 mAh g^−1^), and showed improved rate capability (2149 mAh g^−1^ at 5 C). Interestingly, the lithiophilic groups generated by acid post-treatment of the PDA coating strengthened adhesion to the PAA binder and reduced interfacial and charge-transfer resistances, thereby enhancing apparent Li^+^ diffusivity. Through this mechanism, the acid treated PDA-coated Si anode achieved enhanced cycling stability alongside a markedly higher ICE.

Recently, there has been intensive research on integrating conductive nanocarbons, graphene and carbon nanotubes, alongside PDA coatings on the Si surface to enhance electrical connectivity and mechanical robustness. Wu et al. designed a PDA coated graphite oxide/Si composite (PDA/GO–Si) that effectively mitigated the large volume expansion and SEI instability of Si anodes [28]. The composite structure consisted of ~30–60 nm Si nanoparticles chemically bridged to GO through aminopropyltriethoxysilane (APTES) functionalization, then conformally encapsulated by a ~8 nm PDA layer that served as both a mechanical buffer and SEI-protective coating. The synthesis involved sequential GO preparation via modified Hummers’ method, Si surface amination, GO–Si coupling, and dopamine polymerization in Tris buffer, followed by a mild thermal treatment (180 °C H_2_/Ar) to partially reduce GO, forming conductive reduced GO (rGO). Electrochemically, the PDA/GO–Si anode exhibited an initial discharge/charge capacity of 2903/1974 mAh g^−1^ (68% ICE) and maintained 1300 mAh g^−1^ after 450 cycles at 500 mA g^−1^, while the uncoated GO–Si electrode decayed to 568 mAh g^−1^ under identical conditions. The composite also showed near-100% Coulombic efficiency and excellent rate capability with capacity recovery upon current reversal. The PDA coating enhanced adhesion between Si and GO, lowered charge-transfer resistance, and contributed to a stable SEI formation, preventing crack propagation and even exhibiting a self-healing-like effect during cycling. Despite the innovative PDA–graphene hybrid design, the still low ICE and the use of nanosized Si particles still pose challenges for direct implementation in industrial battery applications.

**Figure 3:**
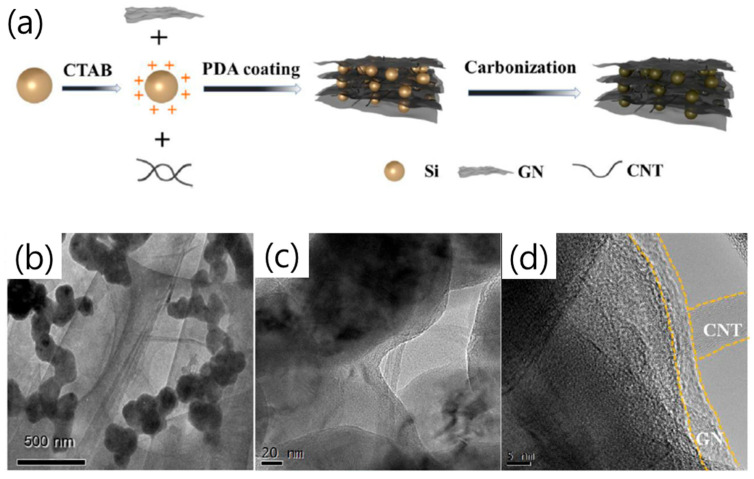
(**a**) Schematic of the synthesis route of C@Si/GN/CNT/PDA-C. (**b**–**d**) TEM images of C@Si/GN/CNT/PDA-C (reproduced with permission from [29], copyright 2022, ELSEVIER).

A recent work reported that combining both graphene and carbon nanotubes with Si particles, together with a PDA coating (C@Si/GN/CNT/PDA-C), enabled high anode conductivity and robust battery stability [29]. The architecture consisted of Si nanoparticles (50–100 nm) modified by hexadecyl trimethyl ammonium bromide (CTAB) to enhance dispersion, electrostatically assembled with graphene and CNTs, and finally encapsulated by a carbonized poly-dopamine (PDA-C) layer (Figure 3a). This coating not only buffered the Si volume change and suppressed direct electrolyte contact but also served as a conductive bridge between Si and GN/CNT, improving structural integrity. Electrochemically, the C@Si/GN/CNT/PDA-C electrode exhibited an initial discharge/charge capacity of 2431/1971 mAh g^−1^ (81.1% ICE), delivering 1946 mAh g^−1^ after 100 cycles at 0.1 A g^−1^ (80% retention) and 1306 mAh g^−1^ after 100 cycles at 1 A g^−1^. The composite also maintained high rate capability (2152, 2020, 1762, 1494, 1220 mAh g^−1^ at 0.1–2 A g^−1^) and rapid capacity recovery, with charge-transfer resistance (R_ct_ ≈ 48 Ω) markedly lower than control samples. A key distinguishing aspect of this work is that CTAB converted the negatively charged Si surface to a positively charged one, thereby improving particle dispersion stability, enhancing interfacial affinity with graphene/CNT, and helping to mitigate Si volume expansion. TEM analyses confirmed uniform PDA-C encapsulation and well-distributed Si within the conductive GN/CNT framework, preventing agglomeration and fracture (Figure 3b–d).

The final dopamine-based design strategy discussed in this review is that integrate bio-derived dopamine with other eco-friendly or synthetic polymers to develop multifunctional binders for Si anodes. Jin et al. proposed a biomass-derived fluorinated corn starch (FCS) emulsion binder for Si and SiOx-based anodes, introducing a sustainable polymeric strategy that enhanced adhesion, flexibility, and interfacial stability in LIB [30]. The binder features fluorine-functional groups and abundant hydroxyl moieties, forming strong hydrogen bonds with the Si surface while improving wettability and SEI uniformity. The FCS polymer was synthesized via fluorination of corn starch using 2,2,3,3,4,4,5,5-octafluoropentyl acrylate, followed by emulsion polymerization. To target Si or Si/SiOx–graphite composite anodes, a physically crosslinked binder network was constructed by simply blending native corn starch, fluorinated corn starch emulsion, and a trace amount of dopamine. Electrochemically, the Si/FCS electrode exhibited an ICE of 86.3%, with a discharge capacity of 1775 mAh g^−1^ after 100 cycles at 0.5 A g^−1^, outperforming Si/starch (1372 mAh g^−1^) and Si/PAA (1201 mAh g^−1^) electrodes. Rate tests further demonstrated high capacity retention (85.2%) at 2 A g^−1^, with significantly reduced charge-transfer resistance and improved Li^+^ diffusion. The enhanced performance raised from the synergistic effects of fluorine substitution, which promotes LiF-rich SEI formation, and the biopolymer’s strong mechanical resilience, maintaining electrode integrity during volume fluctuations.

**Figure 4:**
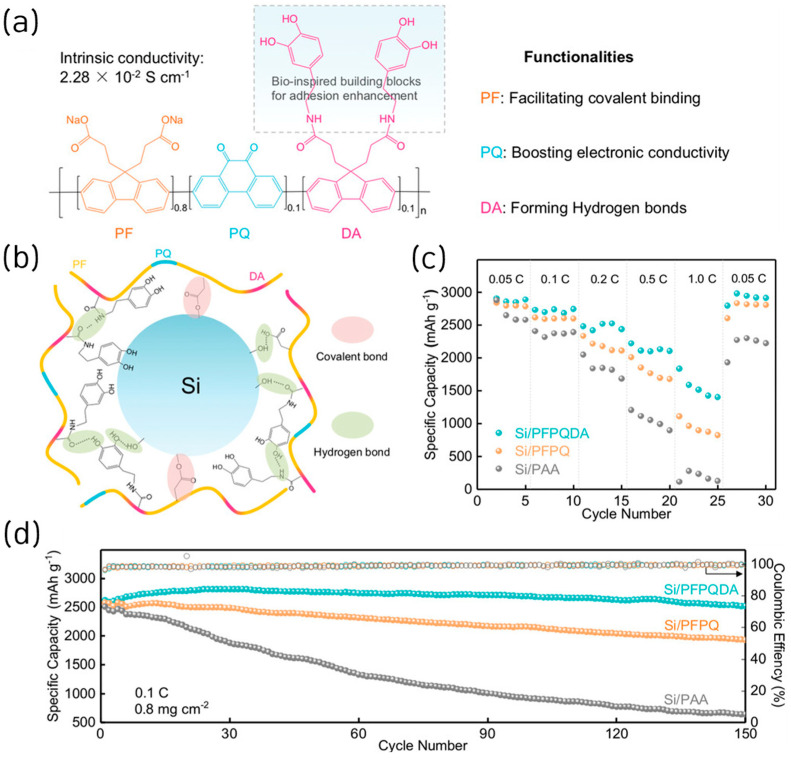
(**a**) Molecular structure of PFPQDA and the illustration of functionalities of each moiety in the monomeric unit. (**b**) Scheme of multiple networks constructed within Si/PFPQDA electrodes. (**c**) Rate performance of Si electrodes using different binders at a current density of 0.05 C, 0.1 C, 0.2 C, 0.5 C, and 1 C. (**d**) Cycling performance of Si electrodes using different binders at a current density of 0.1 C after five cycles at a rate of 0.05 C (reproduced with permission from [31], copyright 2022, Wiley-VCH GmbH).

By integrating dopamine-functionalized fluorene (DA) units into a polyfluorene-phenanthraquinone (PFPQ) copolymer backbone, a bio-inspired conductive polymer binder (PFPQDA) was also developed to achieve a robust conductive network and improved mechanical strength for Si and SiOx anodes [31]. The designed binder introduced multiple polar groups (–COO^−^, –OH, –CONH–) that established hierarchical binding networks, covalent and hydrogen bonds, enhancing adhesion and elasticity within the electrode (Figure 4a,b). The PFPQDA polymer exhibited a high electronic conductivity of 2.28 × 10^−2^ S cm^−1^, nearly identical to PFPQ but nine orders of magnitude higher than PAA. Electrochemically, the ICE of Si electrodes using PFPQDA, PFPQ, and PAA was 72.3%, 72.9%, and 74.3%, respectively. In addition, Si/PFPQDA anodes delivered a specific capacity of 2618 mAh g^−1^ at 0.1 C with 96% capacity retention after 150 cycles, outperforming PFPQ (1930 mAh g^−1^, 75%) and PAA (642 mAh g^−1^, 26%) (Figure 4d). The Si/ PFPQDA electrode also showed excellent rate performance, maintaining 2800 mAh g^−1^ (96.8%) upon current reversal (Figure 3c). The improved performance was attributed to the synergistic covalent and dynamic hydrogen bonding network that suppressed electrode expansion (<17%) and preserved conductive pathways during cycling. The binder’s advantages also extended to micron-scale SiOx electrodes, which retained 80% capacity after 200 cycles at 0.4 A g^−1^, demonstrating scalability and compatibility with industrial materials. A full-cell test with a lithium iron phosphate (LFP) cathode confirmed stable operation (>100 mAh g^−1^ for 60 cycles).

Overall, dopamine-derived materials have demonstrated remarkable versatility in addressing the intrinsic challenges of Si anodes through surface functionalization, conductive carbon conversion, and molecularly engineered binding networks. The evolution from simple PDA coatings to complex hybrid and polymeric systems underscores the adaptability of dopamine chemistry in balancing electronic conductivity, mechanical compliance, and interfacial stability [32]. Despite these advances, challenges remain in optimizing ICE, simplifying synthesis, minimizing dopamine loss during polymerization and carbonization, and ensuring scalability for industrial integration. Continued efforts in rational molecular design and process engineering will be crucial for translating dopamine-inspired strategies into commercially viable silicon anode technologies.

## 3. Self-Healing 3D Network Binders in Multifunctional Silicon Anode Systems

In contrast to direct surface coating of Si particles, the chemical modification of polymeric binders to form 3D network structures has emerged as an effective approach to mitigate the severe volume expansion of Si anodes [33,34]. In recent years, increasing attention has been devoted to the development of self-healing polymer binders functionalized with diverse molecular moieties, which enhance interfacial adhesion between Si particles and current collectors while simultaneously improving the mechanical integrity and electrochemical stability of Si-based electrodes [35]. Self-healing polymers and dynamic crosslinked networks can autonomously repair structural damage and prolong electrode lifetime under repeated cycling, capable of accommodating mechanical strain, maintaining electronic pathways, and stabilizing the electrode–electrolyte interface. This section highlights the recent development of polymer binders engineered with reversible bonding chemistry, bio-inspired adhesion, and conductive frameworks, which collectively contribute to suppressing the pulverization and detachment of Si anodes.

**Figure 5:**
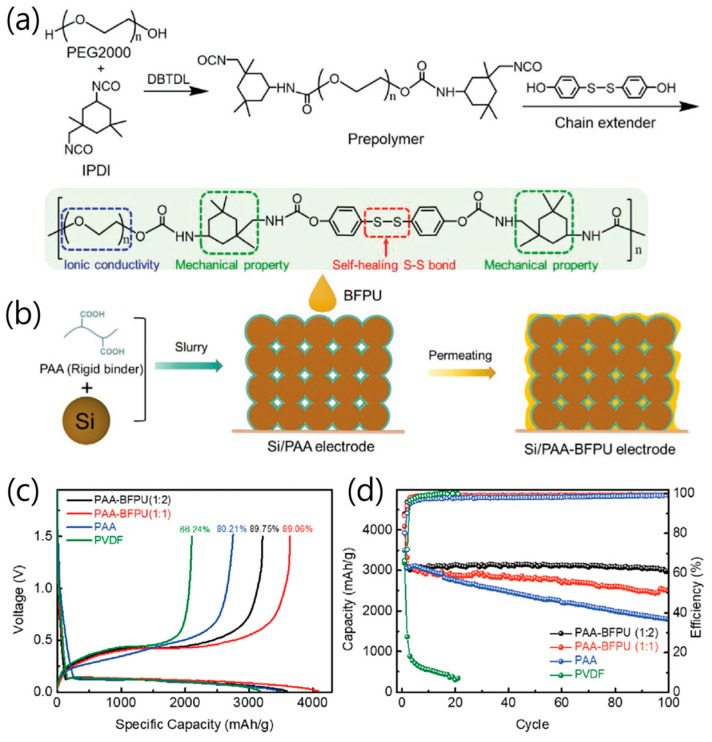
(**a**) Synthesis of the BFPU polymer. (**b**) Fabrication of the Si anode with double-wrapped PAA–BFPU binder. (**c**) Typical charging and discharging voltage profiles of the Si electrode with PAA–BFPU (1:2), PAA–BFPU (1:1), PAA, and PVDF binders at a current density of 0.2 A g^−1^. (**d**) Cycling performance and Coulombic efficiencies of Si electrodes with PAA–BFPU (1:1 and 1:2, respectively, weight ratio), PAA, and PVDF binders at a current density of 1.2 A g^−1^ (reproduced with permission from [36], copyright 2021, Wiley-VCH GmbH).

Jiao et al. proposed a double-wrapped binder design combining a high-modulus PAA inner layer and a low-modulus bifunctional polyurethane (BFPU) outer layer to achieve stress-dissipative, self-healing functionality in Si anodes for LIB (Figure 5a) [36]. The rigid PAA dissipated lithiation-induced stress through strong adhesion to Si particles, while the elastic BFPU layer buffered residual stress and dynamically recovered cracks via reversible disulfide bond exchange (Figure 5b). The two polymers were covalently cross-linked through ester bonds, creating a gradient “hard-to-soft” binder architecture that maintained electrode cohesion during large volume fluctuations. Electrochemically, the Si/PAA–BFPU (1:2) electrode delivered an ICE above 89% and achieved 97% capacity retention after 100 cycles at 1.2 A g^−1^, outperforming Si/PAA (60%) and Si/PVDF (0%) (Figure 5c). Even at a high Si loading (1.54 mg cm^−2^), it maintained 86% capacity after 100 cycles, and exhibited excellent rate capability (1309 mAh g^−1^ at 6 A g^−1^) (Figure 5d). Finite element simulations confirmed that the gradient binder configuration effectively distributed stress within the electrode, preventing Si particle fracture. This work established a mechanically rational binder framework where synergistic rigid–elastic coupling enabled energy dissipation, rapid self-healing, and structural resilience, providing an important pathway toward long-life, high-energy-density Si-based anodes.

A self-healing polymer binder for Si anodes based on dynamic carbon radicals within a cross-linked PAA matrix functionalized with diarylbibenzofuranone (DABBF) was reported, which undergoes reversible C–C bonding/debonding at room temperature to autonomously repair structural damage [37]. The synthesized xPAA-DABBF binder formed a 3D crosslinked network through esterification between the hydroxyl groups of DABBF and carboxyl groups of PAA, thereby enhancing mechanical strength, elasticity, and adhesion to Si particles. The optimized composition (xPAA-DABBF-2.5) achieved the highest adhesion force (2.11 N cm^−1^) and strong self-healing behavior without external stimuli. Electrochemically, Si@xPAA-DABBF-2.5 exhibited an ICE of 77.9% and retained 1774 mAh g^−1^ after 500 cycles (0.5 C) with 47.7% retention, outperforming Si@PAA (694 mAh g^−1^, 21.4%). The anode also delivered 1831 mAh g^−1^ at 5 C, showing excellent rate capability (Figure 4c). Cyclic voltammetry (CV) and electrochemical impedance spectroscopy (EIS) analyses revealed improved Li^+^ diffusivity (4.5 × 10^−14^ cm^2^ s^−1^ vs. 5.7 × 10^−15^ cm^2^ s^−1^ for PAA) and low SEI resistance (R_SEI_) and charge transfer resistance (R_ct_), attributed to stable SEI formation. Moreover, full-cell tests with NCM811 cathodes demonstrated an ICE of 73.6% and superior cycle stability over PAA-based cells. Overall, this work introduces the first C–C radical-based self-healing binder, combining structural resilience, and reversible crosslinking, representing a promising strategy for durable, high-capacity Si anodes. 

PAA polymer is recognized for their effectiveness in preventing the expansion of Si electrodes, attributable to their high adhesive strength and flexibility. However, despite these advantages, PAA faces several limitations and challenges that require attention. A major drawback of using PAA as a binder for Si anodes is its 1D linear structure, which can easily slip from the surface of the active material. This results in mechanical degradation and the detachment of electrode materials from the current collector. Recent studies have reported the integration of adhesive dopamine onto the PAA binder to solve these problems. This review highlights two innovative studies that stablished a self-healing, 3D interfacial network on Si surface, by chemically coupling PAA binders with dopamine. The first study integrated amino-functionalized Si (Si–NH_2_) with dopamine-modified PAA (PAA–DA), forming a 3D supramolecular network through reversible hydrogen and ionic bonds [38]. This architecture allowed dynamic stress dissipation, strong interfacial adhesion, and improved Li^+^ transport via polar functional groups. 

Electrochemically, the Si–NH_2_@PAA–DA electrode exhibited a reversible capacity of 2160.1 mAh g^−1^ after 100 cycles at 400 mA g^−1^ and maintained 1834.1 mAh g^−1^ after 300 cycles at 2 A g^−1^, outperforming Si@CMC–SBR and Si@PVDF electrodes. It also achieved a high initial Coulombic efficiency of 83.3% and excellent rate performance (2671.6 mAh g^−1^ at 1 C). This study introduces a novel supramolecular binder design paradigm. By covalently bonding bio-inspired dopamine to a PAA polymer binder and incorporating an amine functional group onto the silicon surface, the design offers several merits, including self-healing capability, ionic conductivity, and mechanical resilience for Si-based anode batteries.

The second study was inspired by the multi-antenna adhesion mechanism of Parthenocissus. A biomimetic self-healing binder (PDB) was designed by grafting dopamine and introducing boronic acid (BA) into a PAA binder, thereby enhancing hydrogen bond density and dynamic reversibility [39]. The dopamine provided catechol and amine functionalities that strengthened interfacial adhesion with Si, while BA contributed to reversible hydrogen-bond crosslinking, enabling self-healing at room temperature. Molecular dynamics simulations confirmed that PDB exhibited the highest Si–binder interaction energy and superior adhesion (1.50 N peeling force) compared to PAA (0.79 N) and PAB (1.29 N). 

Electrochemically, the Si@PDB anode achieved an initial capacity of 3786 mAh g^−1^ and ICE of 86.2%, maintaining 2538 mAh g^−1^ after 200 cycles at 0.25 C and 1973 mAh g^−1^ at 2 C. Even at ultra-high loading (3.89 mg cm^−2^), it delivered 3 mAh cm^−2^ areal capacity, satisfying practical cell standards. The binder also demonstrated high ionic conductivity (8.53 × 10^−4^ S cm^−1^) and strong electrolyte wettability (contact angle ≈ 17°). SEM and XPS analyses revealed that PDB maintained crack-free electrode morphology and facilitated the formation of a thin, uniform, LiF-rich SEI. In comparison with the previously reported PAA modification approaches, this study offers a simple yet effective synthesis strategy, wherein the incorporation of boronic acid through simple blending imparts enhanced adhesion, dynamic self-healing capability, and a robust 3D network—rendering the design both scientifically meaningful and highly promising for industrial implementation.

**Figure 6:**
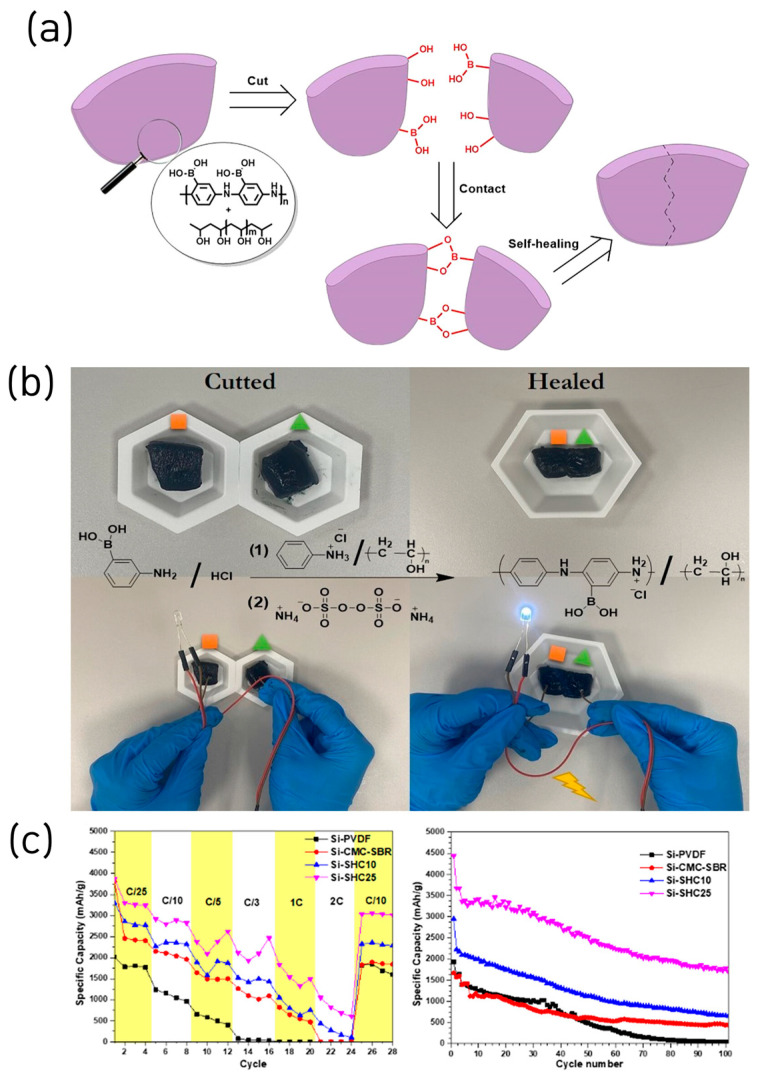
(**a**) Schematic representation of the self-healing mechanism of the SHC binder. (**b**) Illustration of the current flow test for cut and healed SHP. (**c**) Comparison of C-rate results, and galvanostatic charge/discharge test results (reproduced with permission from [40], copyright 2025, ACS).

Very recently, a new binder design was reported in which boronic acid was covalently integrated into the backbone of a conductive polymer, thereby simultaneously achieving exceptional self-healing capability and high electrical conductivity. Yuca et al. introduced a multifunctional self-healing conductive (SHC) binder based on poly(aniline-co-3-aminophenylboronic acid)/poly(vinyl alcohol) for Si anodes in LIB (Figure 6a) [40]. The SHC binder integrated intrinsic self-healing behavior via dynamic hydrogen bonding and electronic conductivity through the polyaniline backbone, effectively addressing the pulverization and conductivity degradation issues of Si electrodes (Figure 6b). The polymer demonstrated autonomous healing within 1 min after mechanical rupture and exhibited excellent elongation and thermal stability. 

Electrochemical analysis revealed that the Si–SHC_25_ electrode (25 wt% binder) achieved a delithiation capacity of 3500 mAh g^−1^ at 0.1C and maintained 1706 mAh g^−1^ after 100 cycles (79.2% ICE). Even at high C-rates (0.5C), the binder sustained electrode integrity, delivering 677 mAh g^−1^ after 200 cycles (Figure 6c). EIS confirmed the lowest charge-transfer resistance (≈30 Ω), while SEM/EDS analyses showed the formation of nanofibrous self-healed regions bridging cracks after cycling, preserving electrode morphology. This study integrates a conductive polymer binder with a self-healing network, an approach we discuss in the next chapter, and points toward forward-looking directions for silicon anode materials.

In summary, polymer-based self-healing binders represent a promising and versatile solution to the mechanical instability of Si anodes. Through the rational design of dynamic covalent and noncovalent bonding motifs, such as hydrogen bonding, disulfide exchange, radical recombination, and boronic acid crosslinking, recent advances have enabled polymers to simultaneously accommodate Si volume changes, maintain electronic pathways, and stabilize interfacial chemistry. The incorporation of bio-inspired molecules, such as dopamine and catechol derivatives, further strengthens adhesion and enhances Li^+^ transport at the electrode–electrolyte interface. Despite these achievements, challenges remain in controlling the molecular architecture, quantifying dynamic bonding strength, and ensuring large-scale process compatibility. Future research should focus on integrating multi-functional chemistries with conductive and elastic frameworks to realize scalable, high-energy-density Si anodes suitable for next-generation LIB.

## 4. Conductive Polymer Binders in Multifunctional Silicon Anode Systems

**Figure 7:**
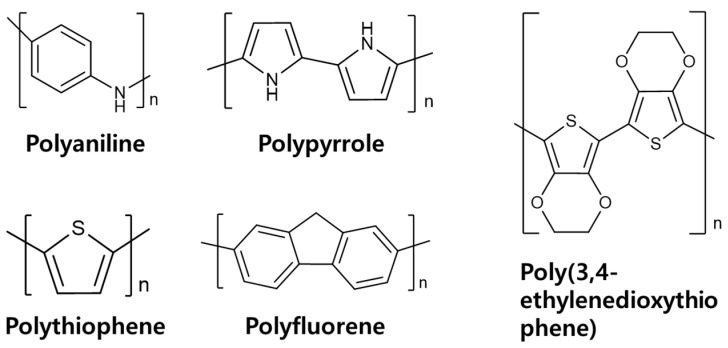
Chemical structures of conductive polymers used in multifunctional Si anodes.

A LIB anode comprises not only lithium-storage active materials (graphite, silicon) but also conductive additives that establish percolated electron pathways and polymer binders that maintain particle cohesion and electrode integrity [41]. Consequently, the dual attributes of viscoelasticity and electrical conductivity make conductive polymers promising one-step replacements for conventional binders and carbon-based conductive additives. In line with this trend, a growing body of S-based LIB research has investigated the use of conductive polymers, including polyaniline, polypyrrole, polythiophene, polyfluorene, and poly(3,4-ethylenedioxythiiophene), as functional components of the anode architecture [42,43]. The chemical structure of the applied conductive polymers are provided in Figure 7. In this chapter, we survey conductive-polymer/Si hybrid configurations and evaluate their electrochemical behavior and practical implications.

**Figure 8:**
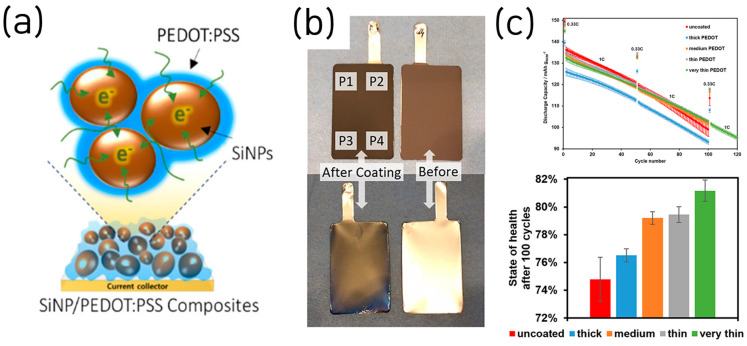
(**a**) Scheme for the experimental PEDOT:PSS/SiNP electrode. (**b**) Photos of a PEDOT-coated compared to a pristine anode from front and back. (**c**) Discharge capacities obtained during the cycling protocol, and corresponding capacity retentions after 100 cycles related to 1 C (reproduced with permission from [45,46], copyright 2016, 2024, ACS).

Given its low cost, electrochemical stability, mechanical robustness, and water-processability, commercially available PEDOT:PSS has been widely adopted as a conductive binder for Si-based LIB anodes [44]. Higgins et al. reported SiNP anode in which a commercial conducting polymer, poly(3,4-ethylenedioxythiophene)/poly-(styrene-4-sulfonate) (PEDOT:PSS), served simultaneously as binder and conductive additive [45]. Homogeneous PEDOT:PSS–SiNP composites with nanoscale sulfur/Si co-distribution were formed, utilizing the polymer’s intrinsic electrochemical stability, mechanical robustness, and electronic conductivity to maintain percolation during Si volume change (Figure 8a). 

Electrochemically, the electrode (≈1 mg cm^−2^) delivered an initial specific capacity 3685 mAh g^−1^ with ~78% ICE and retained the specific capacity of 1950 mAh g^−1^ (~75%) after 100 cycles at 1 A g^−1^; in thicker films (≈1.5 mg cm^−2^) it achieved 3 mAh cm^−2^ areal capacity, supported by polymer conductivities up to 4.2 S cm^−1^ after formic-acid doping. In this work, formic acid served as an in situ secondary dopant for PEDOT:PSS during slurry mixing, inducing chain reorganization that boosted polymer conductivity by ~100× (to ~4.2 S cm^−1^), evaporated upon drying, and, unlike ex situ acid soaks, avoided Cu current-collector corrosion and adhesion loss, thereby simplifying the process.

Stehle et al. also investigated thickness-controlled conductive polymer coatings on Si-dominant anodes, showing that ultrathin PEDOT layers (formed by in situ electropolymerization) improved full pouch-cell cycling stability by mitigating SEI instability and contact loss [46] (Figure 8b). The study used porous, high-loading Si electrodes (≈80 wt% Si; ≈3.0 mAh cm^−2^) uniformly covered by PEDOT, which acted as a flexible, electronically conductive, “artificial SEI” that prevented Si expansion while passivating parasitic reactions at the electrolyte interface. 

Electrochemically, thinner PEDOT layers yielded higher capacity retention, lower direct current internal resistance, and longer life in NCM811//Si full pouch cells (e.g., ~7% higher state of health (SOH) after 100 cycles at 1 C and ~18% longer life to 80% SOH), whereas overly thick coatings reduced ICE/capacity by hindering Li^+^ transport (Figure 8c). This study is oriented toward industrial application, as the authors evaluated PEDOT coatings in full pouch-cell configurations with an areal capacity of 3.0 mAh cm^−2^. Nonetheless, the need to substitute stainless-steel for copper current collectors to prevent Cu corrosion during EDOT electropolymerization, along with position-dependent variations in PEDOT thickness, underscores the need for further process optimization to achieve uniform deposition and compatibility with standard Cu foils.

Polyaniline (PANI) is a conductive polymer widely used in energy-storage applications owing to its broad operating potential window, high stability, and facile synthesis [47]. However, pristine PANI lacks functional groups that strongly anchor to silicon particles; accordingly, strategies typically introduce such moieties via copolymerization or doping to enable Si–polymer covalent bonding or strong hydrogen bonding. The copolymerization approach formed a layered, conductive polyaniline (LCP) coating with an in-situ formed hybrid SEI to stabilize Si anodes by simultaneously improving electronic/ionic transport and interfacial robustness [48]. The approach chemically bridged Si to PANI using a trimethoxysilylpropylaniline (TMSPA), followed by tungstic acid–assisted self-assembly to create a conformal LCP shell that resulted in the uniform SEI formation. 

Electrochemically, half cells showed an initial CE of ~76% that quickly reaches 99%, rate capability up to 942 mAh g^−1^ at 5 A g^−1^, and 1000 mAh g^−1^ after 300 cycles at 1 A g^−1^. Full cells (NCM811, N/P ≈ 1.1) delivered ~2 mAh cm^−2^ after ~45 cycles. XPS depth profiles reveal that Li_2_O and LiF intensities were almost unchanged across sputter depth, implying a fairly uniform inorganic SEI distribution. Nevertheless, a relatively low ICE of 76% is reported, likely arising from reactions at the electrolyte/LCP interface. To mitigate this limitation, the authors further evaluated battery performances using larger Si microparticles and higher areal loadings, demonstrating stable cycling and thereby strengthening the practical applicability of the LCP coating approach.

**Figure 9:**
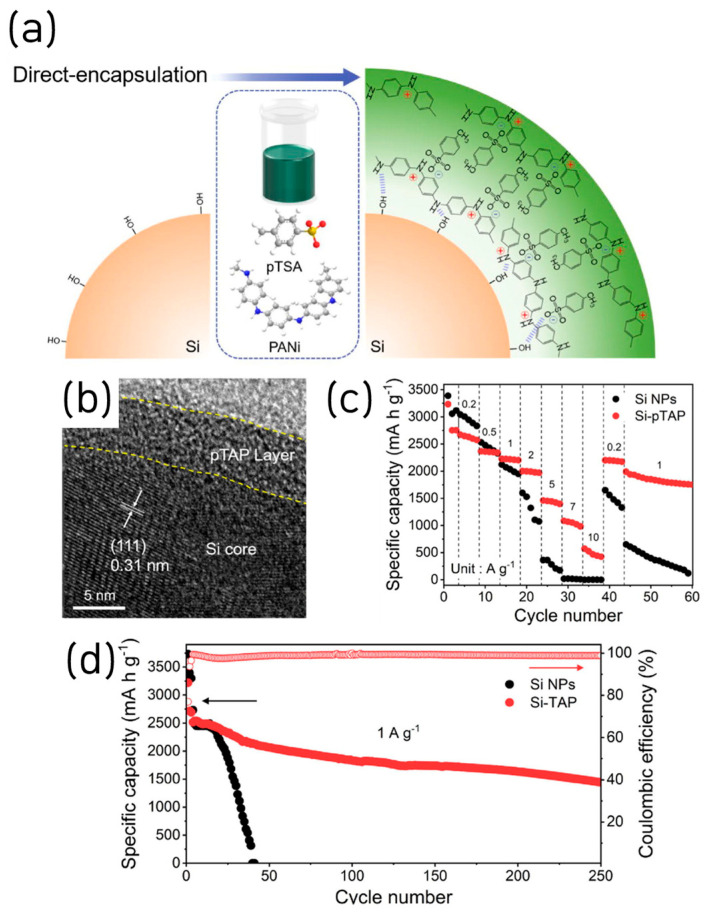
(**a**) Schematic of Si surface modified via direct encapsulation of Si-pTAP synthesis assisted by its enriched hydrogen bonding. (**b**) HR-TEM image of the Si-pTAP. (**c**) Rate capability and (**d**) long-term cycling performance of Si NPs and Si-pTAP (reproduced with permission from [49], copyright 2024, ELSEVIER).

Next, we highlight a recent paper in which doping-engineered PANI achieved significantly improved interfacial coupling to Si [49]. This study introduced a lamellar *p*-toluenesulfonic-acid doped polyaniline (pTAP) nanolayer as an electrolyte-compatible, conductive artificial SEI for Si anodes, formed by facile direct encapsulation via hydrogen-bonding to silanol groups (Figure 9a). Structural/chemical analyses confirm a conformal ~5 nm coating that stored Li^+^ in interlayers and promoted uniform, LiF-rich SEI formation under FEC-containing electrolytes (Figure 9b). Kinetically, EIS over cycling showed markedly lower R_SEI_/R_ct_ and a steeper Warburg slope, while CV/galvanostatic intermittent titration technique (GITT) yielded higher apparent Li^+^ diffusivity across lithiation/delithiation. 

Electrochemically, the pTAP-coated Si delivers high-rate capability (570 mAh g^−1^ at 10 A g^−1^) and long life (>1430 mAh g^−1^ at 1 A g^−1^ after 250 cycles) (Figure 9c,d). The ICE was 78%; however, it increased over the SiNPs after the following cycling, and the CE rapidly rose to over 99% after two further cycles. This study exhibits strong industrial relevance by employing a simple PANI-doping protocol that enhances electronic conductivity and interfacial coupling to Si. In addition, it systematically elucidates the formation of an artificial SEI via conductive-polymer coating through comprehensive structural, chemical, and physical characterization, yielding valuable academic insights.

Following the PANI-doping work, a study that employed PANI in concert with poly(ethylene glycol) (PEG) to mitigate Si swelling and engineer an electrolyte-compatible SEI layer was highlighted [50]. The paper introduced a bi-directional H-bonding modulated PEG–PANI nanolayer as an electrolyte-compatible, conductive artificial SEI for SiNPs, formed by a simple solution process (PEG and PANI co-encapsulation in NMP, solvent removal). Structural/chemical analyses confirmed a uniform ~10 nm PP coating (PEG alone ~3 nm) tightly bound to Si via PEG–Si and PEG–PANI H-bonding. DFT calculation also showed stronger adsorption between PP nanolayer and Si than between pure PANI and Si. 

Electrochemically, the PEG–PANI coating yielded high ICE (≈90.5%), long life (≈1871 mAh g^−1^ after 100 cycles at 1 A g^−1^; 85.7% retention), strong rate capability (≈1955/1333 mAh g^−1^ at 4/8 A g^−1^), and stable operation under a fixed-capacity protocol (≈500 cycles at 1000 mAh g^−1^). High-loading electrodes (≈2.36 mg cm^−2^) retained 3.01 mAh cm^−2^ after 100 cycles at 0.5 A g^−1^, and NCM622//Si@PP full cells (N/P≈1.1) delivered 3.13 → 1.9 mAh cm^−2^ over 50 cycles at 0.2 C with consistent voltage profiles. This paper employs PEG and PANI as soft/hard-modulated coating layers that jointly suppressed Si volume expansion, established continuous ionic/electronic conductive channels, and promoted FEC reduction to yield a LiF-rich, mechanically robust SEI. Furthermore, replacing carbon black with a conductive polymer binder yielded high performance, reinforcing the promise of conductive polymer binders for practical LIB. 

In addition to commercially prevalent PEDOT:PSS and PANI, numerous conductive polymers have been explored as anode components for Si-based LIBs. Since polypyrrole (PPy) offers superior conductivity compared to PANI (100–400 S cm^−1^, facile synthesis, and good chemical stability, it has been explored for Si-based LIB anodes. Nevertheless, PPy does not intrinsically bear Si-affinitive functionalities; accordingly, studies have adopted wet-chemical deposition onto spongelike Si scaffolds to strengthen the Interface [51]. On the other side, polythiophene (PTh) has long been used across diverse applications, including solar cells, organic semiconductors, and sensors owing to its outstanding electronic properties [52]. Because polythiophene is also intrinsically hydrophobic and possesses a rigid backbone, its application to silicon-based anodes typically requires polymer-chemistry modifications; reported strategies include copolymerization with flexible polymers and the introduction of hydrophilic functional groups onto the thiophene backbone [53,54]. As discussed above, effective use of conductive polymers in Si-based anodes, whether as conductive network, binder, or artificial SEI coating layer, generally necessitates extra synthetic tailoring (e.g., copolymer design, introduction of targeted functional groups). At the same time, multiple constraints must be addressed, including preserving electrical conductivity, ensuring solvent solubility and dispersion, and enhancing electrochemical stability. Table 1 summarizes the key performance parameters of various polymer-coated Si anodes.

## 5. Conclusions

In this review, we have discussed the recent progress in polymer-enabled strategies for stabilizing Si anodes, emphasizing dopamine-derived chemistries, self-healing 3D binder networks, and conductive polymer binder systems. Research on bio-inspired dopamine has introduced several routes, including dopamine coatings on Si surface by using its sufficient functional groups, carbon interlayer forming by carbonization, and interfacial bond strengthening by post-treatment. Building on the distinctive adhesiveness of dopamine moieties, recent studies have introduced conductive pathways by co-integrating nanocarbons (carbon nanotubes, graphene) or by hybridizing dopamine with conductive polymers. We also investigated a 3D network architecture, achieved by introducing various self-healing moieties into the PAA binder, that mitigates Si volume expansion and suppresses electrode delamination. Finally, we reviewed studies that enhance interfacial coupling with Si via doping of conductive polymers and copolymer design. Collectively, these strategies delivered high reversible capacities, improved ICE, robust rate performance, and outstanding cycling stability of Si anodes in LIBs. These advancements, supported by the comparative metrics in Table 1, are particularly evident in terms of interfacial stability and mechanical resilience. Regarding interfacial stability, strategies like PEG-PANI have effectively addressed the low ICE in Si anodes by minimizing irreversible Li^+^ loss through polymer-induced SEI formation (achieving an ICE up to 90.5%) [50]. Furthermore, improvements in mechanical resilience are exemplified by self-healing 3D network binders, which demonstrate a superior ability to dissipate stress and autonomously repair cracks during cycling (e.g., 97% retention after 100 cycles) [36].

Looking forward, polymer frameworks for Si anodes should satisfy a set of requirements: strong adhesion to Si and the current collector; mechanical robustness with elastic compliance; chemical/electrochemical stability over a wide potential window; self-healing to restore cracks and contact; and high electrical conductive for electronic/ionic transport. These attributes not only mitigate pulverization and delamination but also stabilize the SEI and reduce internal resistance. However, meeting all of these requirements with a single polymer binder or a single previously described system remains challenging. Therefore, it is necessary to develop a new polymer architecture that combines strong dopamine-based interfacial bonding [55,56], a resilient self-healing 3D network structure, and high electronic conductivity provided by conductive polymers [57,58,59] and nanocarbons [60,61,62].

**Figure 10:**
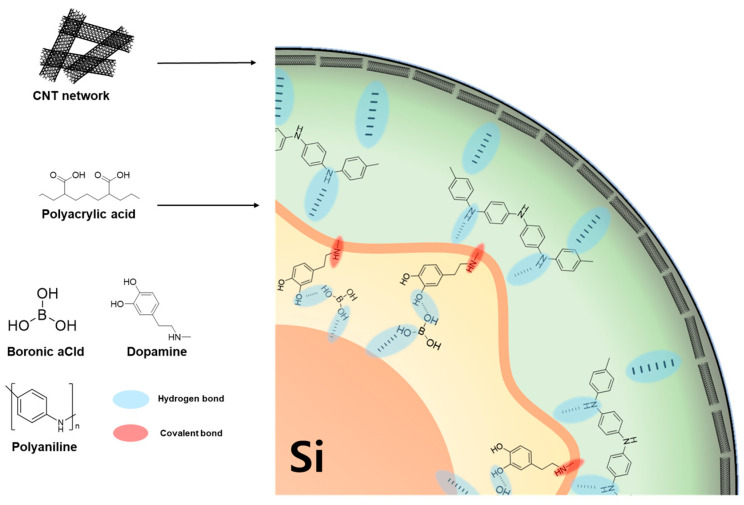
Proposed ideal polymer coating system for the Si anode in LIB.

Guided by these principles and the schematic design space (Figure 10), we propose a multi-layered polymer architecture designed to bridge the gap between academic research and industrial requirements. Specifically, this system should integrate (a) an inner “anchoring” interphase that forms robust chemical and dynamic bonds to native Si/SiOₓ (functionalized with catechol or boronate) for the reduction in interfacial resistance and prevention of delamination from the Si surface; (b) a mid-layer “stress-dissipating” network with abundant H-bond/ionic sites to shuttle Li^+^ and to accommodate >300% volume expansion via self-healing mechanisms; and (c) an outer percolated “conductive skeleton” (conjugated polymer, graphene/CNT framework) to maintain through-thickness electron pathways, acting as an artificial SEI to block electrolyte decomposition [63]. A gradient “soft-to-hard” architecture, validated in recent two-layer systems, exemplifies how stress can be redistributed without sacrificing conductivity. By synergistically combining these functionalities, such an architecture is predicted to achieve an ICE exceeding 92% and retain >80% capacity over 500 cycles even at commercially relevant areal capacities (≥3.0 mAh cm^−2^), overcoming the current trade-off between mechanical stability and electrochemical reversibility.

We hope this review will serve as a practical guideline for designing new polymer coating systems for Si-based LIB anodes by clarifying structure–process–performance relationships, defining realistic performance targets, and outlining manufacturable routes to device-level integration.

## Figures and Tables

**Table 1:** Comparison of the key LIB performance parameters among various polymer-coated Si anodes.

Key Features	Initial Capacity (mAh g^−1^)	ICE (%)	Electrode Loading (mg cm^−2^)	Cycling Stability (mAh g^−1^)	Retention Rate (%)	C-Rate Condition	Ref.
SiOx@N-C (Polydopamine-derived N-doped carbon coating)	2472/1610	65.1	1.0	1514 after 100 cycles	85	0.1 A g^−1^	[25]
Si@PD-x-PAA (PDA wrapped Si)	3600/2600	68.9	0.5	~1800 after 100 cycles	72	1.5 A g^−1^	[26]
Si@C-AT (acid-treated)	3891/3309	85.7	0.75–0.85	1575 after 200 cycles	50	0.5 C	[27]
PDA@(rGO-Si)	2903/1974	68	N/R	1300 after 450 cycles	≈65.9	0.5 A g^−1^	[28]
C@Si/GN/CNT/PDA-C	2431/1971	81.1	1.3–1.5	1946 after 100 cycles	80	1.0 A g^−1^	[29]
Si/FCS (Fluorinated corn starch emulsion, dopamine-assisted network)	3801/3300	86.3	0.7–1.0	2874 after 100 cycles	88	1.0 A g^−1^	[30]
Si/PFPQDA (Conductive binder with dopamine-functionalized)	3700/2675	72.3	0.8	2618 after 150 cycles	96	0.1 C	[31]
Si/PAA-BFPU Bifunctional Polyurethane	3900/3500	89	1.0	97% after 100 cycles	97	1.2 A g^−1^	[36]
SiNP/Cross-linked PAA-DABBF	3900/3050	77.9	0.45–0.5	1774 after 500 cycles	47.7	0.5C	[37]
Si–NH_2_@PAA–DA	3195.5/2661	83.3	1.0	2160.1 after 100 cycles; 1834 after 300 cycles	51.3	0.4, 2 A g^−1^	[38]
Si/PDB (PAA-Dopamine + Boric Acid)	3786/3263	86.2	0.9	2538, 1973 after 200 cycles	67	0.25, 2 C	[39]
Si/P(ANI-co-3-aminophenylboronic acid)/PVA	4125/3500	79.2	0.8	1706 after 100 cycles; after 200 cycles	30.1	0.1, 0.5 g^−1^	[40]
SiNP/PEDOT:PSS	3685/2854	78	1.0	1950 after 100 cycles	75	1.0 A g^−1^	[45]
PEDOT-coated Si (Electropolymerization, thickness-controlled)	N/R	>80	1.48–1.52 mgSi cm^−1^	118 in NCM 811 full cell after 100 cycles	78	1 C	[46]
SiNP@ Layered Conductive Polyaniline	2900/2200	76	1.0	>1000 after 300 cycles;	70	1 A g^−1^	[48]
SiNP@pTAP	3250/2550	78	1.0	>1430 after 250 cycles	63.6	1 A g^−1^	[49]
SiNP@PEG-PANI	2410/2184	90.5	0.8–1.0	1871 after 100 cycles	85.7	1 A g^−1^	[50]

## Data Availability

No new data were created or analyzed in this study. Data sharing is not applicable to this article.
